# Rabies trend in China (1990–2007) and post-exposure prophylaxis in the Guangdong province

**DOI:** 10.1186/1471-2334-8-113

**Published:** 2008-08-21

**Authors:** Han Si, Zhong-Min Guo, Yuan-Tao Hao, Yu-Ge Liu, Ding-Mei Zhang, Shao-Qi Rao, Jia-Hai Lu

**Affiliations:** 1 Department of Medical Statistics and Epidemiology, School of Public Health, Sun Yat-Sen University, Guangzhou 510080, PR China; 2 Laboratory for Tropical Disease Control and Prevention (Key Laboratories of the Ministry of Education of China), Sun Yat-Sen University, Guangzhou 510080, PR China; 3 Centre for Experimental Animal, Sun Yat-Sen University, Guangzhou 510080, PR China; 4 Departments of Molecular Cardiology and Cardiovascular Medicine, the Cleveland Clinic Foundation, 9500 Euclid Avenue, Cleveland, OH 44195, USA

## Abstract

**Background:**

Rabies is a major public-health problem in developing countries such as China. Although the recent re-emergence of human rabies in China was noted in several epidemiological studies, little attention was paid to the reasons behind this phenomenon paralleling the findings of the previous reports. The purpose of this study is thus first to characterize the current trends of human rabies in China from 1990 to 2007, and then to define better recommendations for improving the post-exposure prophylaxis (PEP) schedules delivered to rabies patients.

**Methods:**

The most updated epidemiological data for 22527 human rabies cases from January 1990 to July 2007, retrieved from the surveillance database of reportable diseases managed by the Ministry of Health of China, were analysed. To investigate the efficiency for the post-exposure treatment of rabies, the details of 244 rabies patients, including their anti-rabies treatment of injuries or related incidents, were ascertained in Guangdong provincial jurisdiction. The risk factors to which the patients were predisposed or the regimens given to 80 patients who received any type of PEP were analysed to identify the reasons for the PEP failures.

**Results:**

The results from analysis of the large number of human rabies cases showed that rabies in China was largely under control during the period 1990–1996. However, there has been a large jump in the number of reported rabies cases since 2001 up to a new peak (with an incidence rate of 0.20 per 100000 people) that was reached in 2004, and where the level has remained until present. Then, we analysed the PEP in 244 rabies cases collected in the Guangdong province in 2003 and 2004, and found that 67.2% of the patients did not seek medical services or did not receive any PEP. Further analysis of PEP for the 80 rabies patients who received any type of PEP indicated that almost all of the patients did not receive proper or timely treatment on the wounds or post-exposure vaccination or rabies immunoglobulins.

**Conclusion:**

While the issue of under-reporting of rabies in previous years may well be a factor in the apparent upwards trend of human rabies in recent years, the analysis of PEP in the Guangdong province provides evidence that suggests that the failure to receive PEP was a major factor in the number of human cases in China. Thus, the data underline the need for greatly improved availability and timely application of high-quality anti-rabies biologicals, both vaccines and immunoglobulins, in the treatment of human bite victims. Controlling dog rabies through pet vaccination schemes may also play a huge role in reducing the rate of human exposure. Education of the public, health care staff and veterinarians will also help to change the current situation.

## Background

Rabies is a viral disease that may affect the central nervous system of any species, but only circulates in mammals [1]. Rabies virus is mainly passed from animal to animal or animal to human through bites or scratches. In addition the virus can also be transmitted by the contamination of wounds. Under very exceptional circumstances, the virus can cross mucous membranes when the patient inhales aerosol [2,3]. Rabies epizootics may be divided into two interrelated cycles, urban and sylvatic. The red fox (*Vupes vulpes*) is one of major vectors of the disease and is it reservoir for sylvatic rabies in Eurasia and in parts of America, but it is not the most frequent risk for transmitting rabies virus directly to humans [3,4]. The more serious rabies risk to human is imposed by urban rabies. The domestic dog plays a principal role as a reservoir and transmitter of urban rabies to humans in China [5]. Humans are also at risk from affected domestic animals or pets such as cattle and cats at large, or wild animals such as the raccoon dog in Eurasia and different terrestrial or flying mammals in the New World [4,6,7]. Moreover, direct human-to-human transmission has been observed [8]. There is no effective treatment after the onset of the associated clinical symptoms. Therefore, the currently recommended intervention strategy is to remove and neutralize the infectious virus before it enters the nervous system [2].

According to the official World Health Organization (WHO) data [9-11], more than 2.5 billion people are at risk in over 100 countries reporting the disease. Rabies has the tenth highest mortality of all infectious diseases worldwide. There are still about 50000 to 60000 human deaths annually although effective vaccines for post-exposure treatment are available [12]. Developing countries account for almost all of the reported human deaths, and most affected are the tropical countries or regions in Africa, Asia, South America and Oceania. During the period 1993–2002, the countries of the Americas reported a decrease of 82% in the number of human cases, with cases plummeting from 216 in 1993 (mortality rate of 0.03 per 100000 inhabitants) to 39 in 2002 (mortality rate of less than 0.01 per 100000 inhabitants) [13]. Rabies is considered as a source of economic loss and, above all, hampers the movement of animals between different countries or regions, which has serious implications for the 'open market' since some countries are currently rabies free and wish to maintain their disease-free status [3].

Rabies is a major public-health problem in most of the developing world [9,14-16]. Prophylactic measures taken in the past, such as destroying foxes and reducing dog populations, did not prevent the spread of the rabies, although recently developed genetically modified rabies virus vaccines provide an effective method of prevention of rabies virus infection in dogs, foxes and raccoons [17-19]. During recent years, most research into the control of rabies has concentrated on the development of post-exposure prophylaxis (PEP) of rabies [3]. The use of human rabies immunoglobulin (HRIG) and of equine rabies immunoglobulin (ERIG) has saved the lives of countless patients who would have died if treated with vaccine alone. However, both products are often in short supply worldwide and virtually unaffordable in developing countries [20]. Therefore, the high demand for PEP in Africa and Asia exerts a substantial economic burden, not only as a result of the high costs of human vaccine and rabies immunoglobulin (RIG) products, but also because of considerable indirect (patient) costs associated with travel and income loss for PEP [21]. Additional economic losses relate to livestock deaths, which, although poorly quantified, may be significant, with an estimated annual incidence of 5 deaths per 100000 cattle, costing US$12.3 million annually in Africa and Asia. The total (direct and indirect) cost of PEP accounts for 5.8% of annual per capital gross national income in Africa (US$40 per treatment) and 3.9% (US$49 per treatment) in Asia [22].

In China, over a 55-year period between 1950 and 2004, 108412 human rabies cases were reported with three major epidemics occurring during this period [23,24]. The first epidemic outbreak occurred in the mid-1950s when cases rose to a peak of about 2000 annually. After a decline in the 1960s, the number of cases again started to increase in the early 1970s reaching a peak in 1982, and then remained at the level of 5000–6000 cases per year until the end of the decade [25]. Therefore, one of the purposes of this study was to conduct a comprehensive analysis of the rabies situation in the country using all of the official data to characterize the current epidemiological trends of rabies in China from 1990 to 2007. In order to define better recommendations for improving the PEP schedules delivered to patients, we also analysed the reasons for the post-exposure treatment failures (or the absence of PEP), based on the medical records of anti-rabies treatment of injuries or related incidents for 244 rabies patients, ascertained in Guangdong province of China in the years of 2003 and 2004.

## Methods

### Data collection

The epidemiological data for 22527 human rabies cases from January 1990 to July 2007 were obtained from the surveillance database of reportable diseases managed by the Ministry of Health of China. The rabies diagnosis in humans reported to the national surveillance data bank was based on the clinical criteria set by the Ministry of Health of China including the history of animal bite(s), intense anxiety, nervousness, paralysis in the area of the bite, hydrophobia and final death. The history of animal bite was confirmed by subsequent case epidemiological surveys from various provincial Centres for Disease Control and Prevention (CDC) offices. To investigate the efficiency for the post-exposure treatment of rabies, 244 rabies patients with their detailed medical records of anti-rabies treatment of injuries or related incidents, enrolled at Guangdong province in the years of 2003 and 2004, were ascertained from all of the reported rabies cases (441 patients) during the periods. Clinical evaluations were obtained from all of the participants (or their relatives) according to the protocols approved by the collectors' institution review boards of the ethics committees.

### Schedule table

The patient's record form adopted for anti-rabies treatment was a standard form designed by the Ministry of Health of China. At the CDC offices, the form was completed by the staff who were responsible for clinical evaluation and treatment. The patients or their responsible parties supplied the information entered into the forms. The variables taken into account for an affected patient were: (a) the patient's demographic profile: place of residence, age and sex; (b) exposure characteristics: date of event, type of exposure (scratch, lick, indirect contact or bite), site of lesion, number of lesions (single or multiple), type of lesion (superficial or deep); (c) treatment: time lag between exposure and onset of treatment (delay in days), procedure adopted (wound care and medication), type and route of drugs (rabies vaccine, animal antiserum and/or immunoglobulin), number of doses prescribed, number of doses administered, type of professionals who assessed the patient and prescribed or delivered the treatment.

### Data analysis

The distribution and the cumulative number of rabies cases over all provincial administrative regions of China for the period 1990–2007 were investigated. The data were then subjected to statistical analysis, and frequencies were calculated for the categorical variables. Two epidemiological indices, incidence rate and mortality rate, were computed to characterize the infectious disease in China. The incidence rate was the number of new cases of rabies diagnosed or reported during a defined period of time (for example, a year), divided by the number of persons in a stated population in which the cases occurred, expressed as cases per 100000 per annum in this study. The mortality rate was calculated by dividing the number of rabies deaths occurring in the population during the stated period of time (for example, a year), by the number of persons at risk of dying during the period. The rabies-specific mortality rate only covered deaths that were a direct result of the disease and was reported on the basis of 100000 persons in this study. To analyse the risk factors that the patients were predisposed to or the regimens given to 244 patients, in an attempt to identify the reasons for the PEP failures or the absence of PEP, a McNemar test, as implemented in a public server [26], was used to test two studied proportions due to the different exposures or factors obtained from the 244 patients. This test took into account the correlation between the two sets of the same patients, occurring because the patients received alternative exposure 1 only, alternative exposure 2 only or neither exposure.

## Results

### Epidemiological characteristics

**Figure 1:**
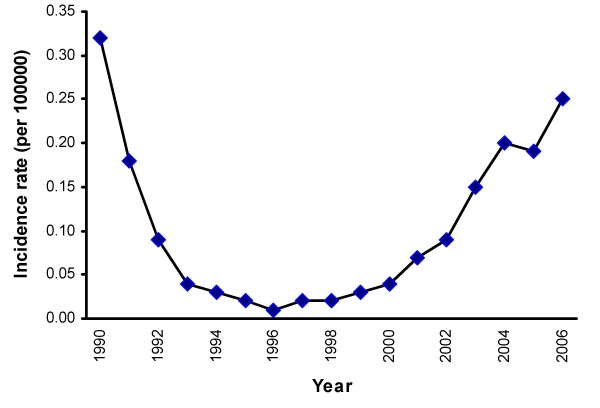
The temporal trends of human rabies in China, from 1990 to 2006.

The annual incidence rate for rabies is summarized in Figure 1. The results from analysis of a total of 22527 human rabies cases from January 1990 to July 2007 showed that rabies in China was largely under control during the period 1990–1996, when nationwide rabies vaccination campaigns were conducted. The data collected showed that after a decrease in human rabies cases during that period, the incidence started to rise and a total of 3279 cases were reported in 2006.

In 1996, the number of reported cases dropped to the lowest frequency (159 cases), in sharp contrast to the figure for 1990, with 3520 cases reported nationwide. The 1996 yearly incidence rate of rabies in China was 0.013 per 100000 inhabitants. During 1996–1999, the yearly incidence rate of rabies, although increasing slightly, was relatively stable, but later the figures jumped dramatically. In 2005, 2571 cases of rabies were documented. Since the start of the new millennium, the incidence rates of human rabies increased from 0.0889 per 100000 inhabitants in 2002 (1159 cases) to 0.1511 (2037 cases) in 2003; this incremental trend continued into 2004 (2651 cases), 2005 (2571 cases) and 2006 (3279 cases). The data for 2007 are incomplete, but between January and July there were 1740 human rabies cases reported, an increase of 28.98% compared with the same period in 2006 (1349 cases).

**Figure 2:**
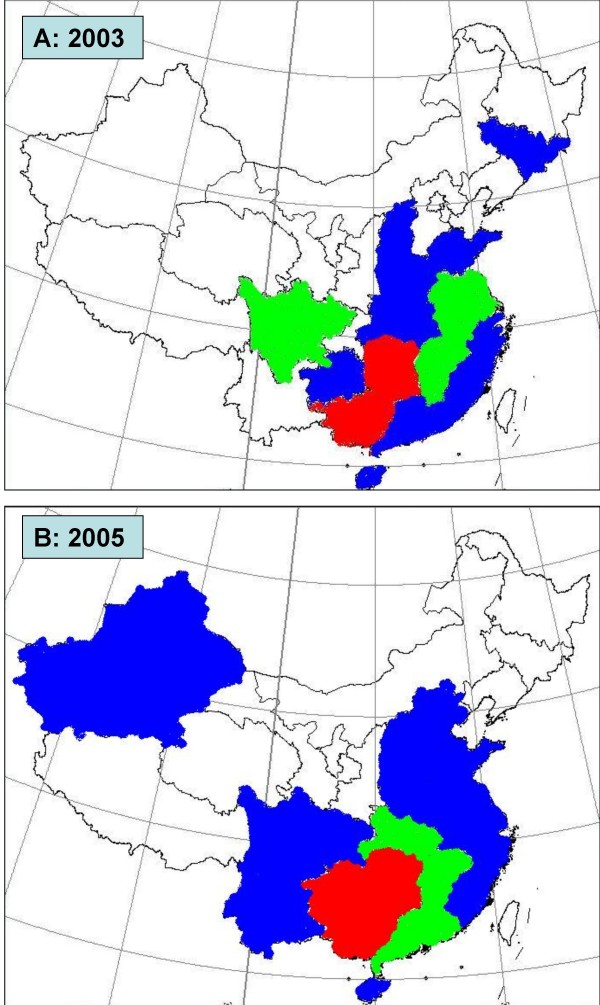
**Geographic distribution of human rabies in China**. Incidence rates in (A) 2003 and (B) 2005 per million people with the scale: blank, none; blue, 0–0.2; green, 0.2–0.4; red, > 0.4.

The incidence of human rabies, however, is not distributed evenly in the vast country of China. Figure 2 shows the geographic distributions in two recent years (Figure 2A for 2003 and Figure 2B for 2005). The highest prevalence in both years was registered in the southwestern and southern territories of China. Hundreds of rabies cases were identified in the regions including Guizhou, Guangxi, Hunan and Guangdong provinces. In 2003, there were no reported cases in the north, northeast or west of China. However, in 2005, human rabies expanded to much wider regions, even the far-west region (Xinjiang provincial jurisdiction; Figure 2B). There were no reported cases in Inner Mongolia, Heilongjiang, Qinghai, Ningxia, Tibet, Gansu or Liaoning provinces. In either year in almost all provinces of China, the mortality rate (data not shown) was identical or similar to the incidence rate as, once clinical signs of rabies appeared, the disease was essentially 100% fatal.

**Figure 3:**
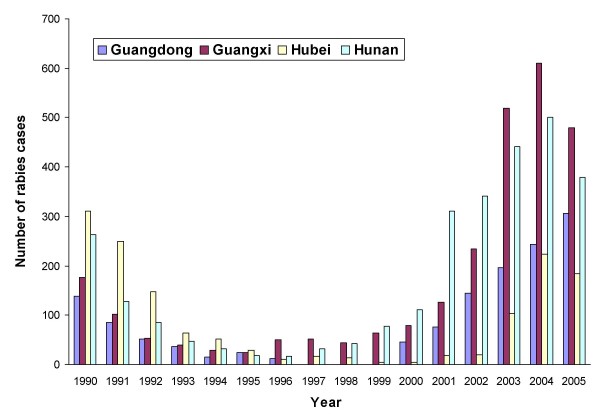
**A comparison of the incidence of rabies between four southern provinces (Guangdong, Guangxi, Hubei and Hunan) of China**. Note that the data for 1997–1999 for Guangdong were not available.

We had a particular interest in four neighbouring, highly populated provinces (Guangdong, Guangxi, Hubei and Hunan) in southern/central China, because the incidence rates were relatively higher, and the corresponding CDCs had more clinical details for the human rabies patients. A comparison between the provinces, shown in Figure 3, demonstrated that Hubei had the highest average number of rabies cases in 1990–1995 (for example, 311 cases in 1990); the number of cases decreased or remained stable until 2000, but there was another large increase from 2001. The same or similar temporal trend of rabies was also observed in the other three provinces, which may well capture the unique epidemiological profiles of the tropical or subtropical southern regions in recent years. These data indicate that while China was largely free of human rabies in the final years of the last century, a worrying trend has started to emerge.

### Post-exposure treatment (PEP)

According to the current WHO guidelines, we divided rabies post-exposures into three categories (see Additional file 1 for details). Exposure category I describes the lightest degree of exposure to infection, without any skin injury, while category III describes the most serious situations where single or multiple transdermal bites or scratches occurred that required immediate wound treatment and anti-rabies vaccines.

China banned nervous tissue vaccines (NTVs) in 1981, so different provinces adopted slightly different options for rabies vaccine products. For example, Guangdong provincial CDC recommended using the following products: purified Vero cell rabies vaccine (PVRV, Aventis Pasteur, Lyon, France), purified chick embryo cell vaccine (PCEV, ChengDa Biologicals, Shengyang, China) and hamster kidney cell vaccine (PHKCV, Lanzhou Institute of Biological Products, Lanzhou, China). Nevertheless, there are substantial numbers of vaccine products produced by small companies or institutes in China, thus lacking suitable quality and efficacy control. These low-quality vaccine products not only increased the difficulties in controlling and preventing rabies, but also complicated the public health programmes in other Asian countries that imported these products. The standard post-exposure vaccination schedule was the 'Essen' 5-dose intramuscular regimen on days of 0, 3, 7, 14 and 28. However, five pre-exposure or post-exposure schedules are currently used in China (see Additional file 2 for details).

### Analysis of post-exposure treatment failures

We analysed the PEP in the rabies cases in Guangdong province. This could be a suggestion that the lack of adequate PEP is one of the major problems in the current situation in China, since Guangdong has one of the highest incidence rates and also has the best information available. There were 197 and 244 human rabies cases reported in 2003 and 2004, respectively. However, only 244 cases (130 cases in 2003 and 114 cases in 2004) had sufficient information (demographic and clinical data) to be suitable for the analysis of post-exposure treatment failures or absence of PEP. To look at the information for the virus transmitters, we found that most of the 244 human rabies cases were infected by dogs (209 cases, 85.7%), followed by cats (9 cases, 3.7%) and rats (6 cases, 2.5%). In detail, the dogs could be classified as owned by the patients themselves (101/209, 48.3%), dogs belonging to some one else in the neighbourhood (38/209, 18.2%), stray dogs (38/209, 18.2%) and others (32/109, 15.3%). A McNemar test revealed that virus transmission by dogs was highly significant (*P *< 0.01) compared with other animals. The frequency of incidents was higher for male patients and most patients were under 20 years old. Direct or indirect contact accounted for 96.47% of the types of exposure and the remaining 3.53% were via unknown means. Per the degrees of exposure described, categories I-III accounted for 33.6%, 38.9% and 22.5% of the patients, and the remaining 5% were not classified due to the incomplete data. The time lags between the incidents and the presentation of patients for anti-rabies assessment ranged from 0 to 3 days in most cases. According to the available data on the lesion sites for 109 patients in 2004, most often affected were the arms (31/109, 28.4%), legs (31/109, 28.4%) or fingers (24/109, 22.0%); see Additional file 3 for a graphical illustration. Using a McNemar test (*P *< 0.01), we found that single injuries (82/109, 75.2%) were more frequent than multiple injuries (18/109, 16.5%).

Among the 244 cases with informative medical records, 67.2% (164/244) did not seek any medical service and the remaining 32.8% (80/244) received PEP. Table 1 shows the analysis of post-exposure treatment failures per the risk factors that patients were predisposed to or regimens given to the 80 patients who received any type of PEP. Among the 80 patients, 62.5% (50/80) only had their wounds washed with water by themselves, 37.5% (30/80) went to hospitals or local CDCs to have proper treatment of their wounds (washed with soap water or clean water for at least 15 minutes, and then embrocated with 2–3% tincture of iodine or 75% alcohol), 45% (36/80) of patients did not receive any rabies vaccine or passive immunization and 47.5% (38/80) of patients received between one and four shots of rabies vaccine, but none of passive immunization. Of the 80 cases who received PEP, only 7.5% (6/80) of patients received a full regime. These six patients had a category III exposure, of which five had a bite on the head and neck and one case had multiple bites. They all received the following treatment within 24 hours of the bites: (1) wounds were washed with soap and water or clean water for at least 15 minutes; (2) wounds were then embrocated with 2–3% tincture of iodine or 75% alcohol; (3) animal antiserum (40 IU/kg) or human immunoglobulin (20 IU/kg) was injected into the area surrounding the wounds; (4) one full ampoule of rabies vaccine was administered IM on days 0, 3, 7, 14 and 28. Five cases received PCEV or PHKCV made in China, and one received PVRV imported from France. Nevertheless, all six patients finally died from the rabies infection. After careful scrutiny of the six cases, the reduced quality of the vaccine due improper storage by the patients themselves after the first shot and lower doses might have contributed to the failures, while the lesion site (on the head and neck or multiple bites) may well be a factor causing the failures in the six cases. In short, according to the current WHO guidelines, none of 244 cases reported received both adequate and sufficient post-exposure treatment.

**Table 1:** Analysis of post-exposure treatment failures per the risk factors predisposed to or regimens given to 80 patients who received any type of PEP, collected in Guangdong province

Factor	Known cases
Multiple wounds and/or bite on head and neck	55 (68.8%)
Insufficient wound treatment	50 (62.5%)
Delay of two or more days	34 (42.5%)
No rabies immunoglobulin given	73 (91.3%)
No rabies vaccines given	74 (92.5%)

## Discussion

In China, human rabies was largely under control in the period 1990–1996, owing to nationwide rabies vaccination programmes. Since the vast majority of cases were a result of canine rabies, an extensive dog vaccination programme was initiated in 1987 [27]. From 1990 to 1996, canine rabies decreased by 95.5%, while the GDP (gross domestic product) increased considerably in the same period. During the period 1996–1999, the yearly trend for human rabies was relatively stable, but the number of human rabies cases jumped dramatically since the start of the new millennium. Overall, our data implicate that while China was largely free of human rabies in the final years of the last millennium, the recent increase in incidence has been high enough to trigger a warning sign for control and prevention.

However, the data ascertained at various CDCs may not well capture the real epidemiological scenarios for human rabies in China. For example, since the SARS (severe acute respiratory syndrome) epidemic of 2003, the Chinese government has set up a systematic and nationwide surveillance network for zoonotic diseases. Increased surveillance together with increased dog or other pet populations may be the principal factors explaining the increasing number of cases of human rabies reported in China in the recent years [27]. The same issues for obtaining unbiased estimates for the epidemiological indices of rabies (incidence or mortality rates) were noted by Torrence et al. [28] in similar studies, and by Brazuna et al. [29] in a study of Brazilians. It should be noted that this bias is not unique to the data ascertained at various CDCs, but is instead common to many medical experiments (for example, hospital-based studies) where random sampling is not a feasible (often less-efficient) strategy for data collection. However, there is a list of factors influencing precise and accurate estimation of human rabies parameters. These include the following factors: (1) many people, especially in remote areas, do not have easy access to a public health service; (2) there was a dearth of knowledge about rabies in the general public and among health workers; (3) there was insufficient diagnostic capability and facilities in the country so that some case reports may not be able to be sent to the surveillance database at the Ministry of Health of China. Despite these limitations, the results clearly demonstrated that rabies constituted a real public problem in China and its control should be a top priority.

Our data also indicate that most incidents occurred in the southwestern and southern territories of China, and most frequently in the highly populated areas. For example, in 2005, rabies was routinely identified in Guizhou (481 cases), Guangxi (480 cases), Hunan (379 cases) and Guangdong (306 cases) provinces. The four rabies-endemic provinces lacked strictly enforced measures to eliminate dog rabies or an ample supply of modern cell culture rabies vaccines for humans. It was also interesting to note that most of patients were young in the range of 0–20 years old and the major victims of rabies were children less than 16 years old, perhaps as they are bitten by dogs more frequently than adults. When attacked by dogs, the lesions were often on the child's head and neck, thus, not surprisingly, this lesion site was associated with the highest risk for developing rabies [21,30,31].

In our study of the 244 human rabies cases in Guangdong province, 67.2% of the patients did not seek medical services or did not receive any PEP. The time lag between the incidents and the presentation of patients for anti-rabies assessment ranged from 0 to 3 days in most cases. Up to 62.5% of the patients did not receive proper treatment of the wounds, 92.5% did not receive adequate post-exposure vaccination and 91.25% did not receive any anti-rabies immunoglobulin. These results suggest that the population investigated may not be aware of the risks of rabies transmission, as revealed previously [23]. This fact could be a significant issue for public health based on the large number of failures for human PEP that have occurred recently in China. During the study, we found that education/information regarding rabies can only be obtained from the public boards at municipal or district CDCs in Guangdong province. We found few such public information boards or web pages at police departments (stations), community hospitals or offices, in villages and so on. In December 2007, we conducted a survey among 270 students of preventive medicine at our institute. We observed that the students had a basic knowledge of rabies, but had some misunderstandings. Among the students, 92.2% could answer questions regarding PEP regimes correctly. However, only 47.4% knew that washing the wounds can remove the virus residue on the lesions, and the percentage of students answering this correctly for students in grades 2–5 was 42.6%, 32.0%, 60.0% and 70.4%, respectively. Only 58.9% of the students knew the pre-exposure administration regime for rabies vaccine. However, 71.5% of the students knew the post-exposure administration regime for rabies vaccine and 57.8% knew the immunization sites for rabies vaccine.

In rabies-infested developing countries, modern cell culture vaccines are too costly for the poorly developed remote regions, so dangerous NTVs are still used [32-34]. Over the last 4 years in China, of the more than 2000 people yearly who died of rabies, only about one-third received rabies vaccinations. This figure was even lower (7.5%) in Guangdong province, as estimated in this study. In most cases of vaccine failures, patients contracted the disease before the full PEP regimen was completed [24]. In addition, there was a critical shortage of human and purified equine rabies immunoglobulin in these regions, which are essential in the treatment of severe exposure. Although the costs of modern vaccines are decreasing, the current price of a full-dose intramuscular vaccine treatment is somewhat beyond what an average family in developing countries can afford [35,36]. For example, the average annual income per capita in Guangdong province is US$3000 and the rabies vaccine costs US$12.5–45.0, taking up 0.42–1.51% of the average annual income per capita. This figure would be much higher we consider the fact that about 60–70% cases were from poor rural areas or habos. Furthermore, the supply of modern and safe vaccines for many provinces are grossly inadequate, whereas the demand for affordable and safe human post-exposure treatment is increasing in these provincial regions [23,24]. Consequently, only anti-rabies vaccines and human immunoglobulin are available, and provide feasible solutions for efficiently controlling rabies in the majority of municipalities of China or other developing countries [16,37-39].

Finally, a mass vaccination dog campaign for the control and elimination of rabies transmitted by dogs is very important. China has not implemented enforced immunization for dogs. Rabies vaccine was provided at the cost of the owners, and the cost was expensive, from tens to hundreds of Chinese Yuan. Dogs were registered at local police departments, while animal rabies vaccines were administrated by veterinarians, thus there was a lack of good communication or effective strategies for rabies control in dogs. It appears that the increase in canine rabies, increase in the dog population and decrease in the vaccine coverage of dogs may have contributed to the apparent upwards trend in human rabies in recent years in China. Based on Chinese CDCs' surveillance of dog rabies in some typical affected areas, the positive rate was 3.9% (4/102, Guangxi province) in 1999, 9.1% (76/838, Hunan, Henan, Guangxi, Guizhou and Jiangsu provinces) in 2004 and 5.9% (5/85, Guizhou province) in 2005. This pattern is fairly consistent with human rabies. Nevertheless, the methods of control undertaken by Latin American countries might provide promising ways for China to develop a more effective programme for controlling human rabies transmitted by dogs. With the support from the Pan American Health Organization, several measures such as the decentralization of health units with PEP available, dog vaccination coverage and dog rabies surveillance have been successful in achieving the goal of eliminating human rabies transmitted by dogs [40].

## Conclusion

This large-scale epidemiological study has demonstrated that human rabies was largely under control in China between the years of 1990 and 1996, via the national rabies vaccination programmes. However, the number of reported rabies cases has started to increase since then. From 2001, this figure increased significantly to a new peak that was reached in 2004, where it has remained. Based on the investigation of 244 human rabies cases collected in Guangdong province in 2003 and 2004, 67.2% of patients did not seek medical services or did not receive any PEP. Further analysis of the post-exposure treatment failures for 80 rabies patients who received any type of PEP indicated that the majority of patients, if not all, did not receive proper and timely treatments on the wounds or post-exposure vaccination. The study implicated that: (1) the incidence of human rabies, which China was largely free from in the final years of the last millennium, is now increasing which should be a warning sign for control and prevention; (2) there is a need to improve the current rabies control programme in order to reduce non-compliance rates and to decrease the occurrence of flaws in the surveillance programme and in the provision of health care. Given the findings of the study, we view the implementation of the following measures as appropriate [9-12,41,42]: (a) continuing supervision of the current human rabies control programme, in order to reduce both non-compliance rates and the occurrence of flaws in health-care provision; (b) improving the interactions between professionals from the divisions of the municipal health-care network and teams of national programmes; (c) increase rabies awareness among Chinese health authorities and policymakers, improve the training of general practitioners and health care workers, and educate school children and the general public, which is crucial for effective rabies control; and (d) urban planning and development should take ecosystem preservation into account, in an attempt to balance the interaction between humans and animals.

## Competing interests

The authors declare that they have no competing interests.

## Authors' contributions

J–HL, HS, Z–MG, and S–QR conceived of and designed this study, and drafted the manuscript. J–HL, HS and S–QR collected the data, and performed the statistical analysis. Z–MG, Y–TH, Y–GL and D–MZ made significant contributions to this work by providing assistance and helped in the data collection, data manipulation and analysis. All authors read and approved the final manuscript.

## Pre-publication history

The pre-publication history for this paper can be accessed here:



## Supplementary Material

Additional file 1 Table S1 – Degree of exposure and treatment schedules for human rabies, adopted from the criteria set by the Ministry of Health of China, 2006. Click here for file

Additional file 2 Table S2 – Pre-exposure and post-exposure schedules for rabies, currently used in China. Click here for file

Additional file 3 Figure S1 – Spatial distributions of rabies classified by the sites of lesion. Click here for file
