# Supplementary material for: Rabies trend in China (1990–2007) and post-exposure prophylaxis in the Guangdong province
Source: BMC Infect Dis. 2008 Aug 21;8:113. doi: 10.1186/1471-2334-8-113 (PMC2532688; doi:10.1186/1471-2334-8-113)
Supplement: Additional file 1 — Table S1 – Degree of exposure and treatment schedules for human rabies, adopted from the criteria set by the Ministry of Health of China, 2006. [file 1471-2334-8-113-S1.doc]

## Table S1 - Degree of exposure and treatment schedules for human rabies, adopted from the criteria set by the Ministry of Health of China, 2006

| Exposure category | Ways of contacting with domestic animals | Degree of exposure/the lesion | Treatment schedule |
| --- | --- | --- | --- |
|  |  |  |  |
| I | 1. get in touch with or feed animals | light | no treatment was necessary if without history of the disease |
|  | 2. intact skin was licked |  |  |
|  |  |  |  |
| II | 1. naked skin was bitten slightly | superficial | wound treatment was immediately given, and then the patient was immunized by human rabies vaccines |
|  | 2. slight scratch or licking was seen, but without obvious haemorrhage |  |  |
|  |  |  |  |
| III | 1. single or multiple transdermal bites or scratches was present | deep | wound treatment was immediately given, and then the patient was injected with both human rabies vaccines and anti-rabies immunoglobulin |
|  | 2. mucous tissue was contaminated by animal body fluid |  |  |
|  | 3. the lesion was licked |  |  |
