# Supplementary material for: Rabies trend in China (1990–2007) and post-exposure prophylaxis in the Guangdong province
Source: BMC Infect Dis. 2008 Aug 21;8:113. doi: 10.1186/1471-2334-8-113 (PMC2532688; doi:10.1186/1471-2334-8-113)
Supplement: Additional file 2 — Table S2 – Pre-exposure and post-exposure schedules for rabies, currently used in China. [file 1471-2334-8-113-S2.doc]

**Table S2 - Pre-exposure schedules and post- exposure schedules for rabies currently used in China**

| **Schedule** | **Description** |
| --- | --- |
| PEP 1 | pre-exposure schedule: using PVRV on days 0, 7 and 28. |
| PEP 2 | post-exposure schedule (Essen schedule):one full ampoule (PVRV, PHKCV or PCECV) was administered IM on days 0, 3, 7, 14 and 28, with/ without giving rabies immunoglobulin (RIG). |
| PEP 3 | post-exposure schedule: one full ampoule (PVRV, PHKCV or PCECV) was administered IM on days 0 and 3 if exposure occurred once again during next 1 year. |
| PEP 4 | post-exposure schedule: one full ampoule (PVRV, PHKCV or PCECV) was administered IM on days 0, 3 and 7 if exposure occurred once again during next 1 to 3 years. |
| PEP 5 | post-exposure schedule: one full ampoule (PVRV, PHKCV or PCECV) was administered IM on days 0 3, 7, 14 and 28 if exposure occurred once again beyond next 3 years. |
