# Supplementary figures and images for: Rabies trend in China (1990–2007) and post-exposure prophylaxis in the Guangdong province
Source: BMC Infect Dis. 2008 Aug 21;8:113. doi: 10.1186/1471-2334-8-113 (PMC2532688; doi:10.1186/1471-2334-8-113)

## Slide 1
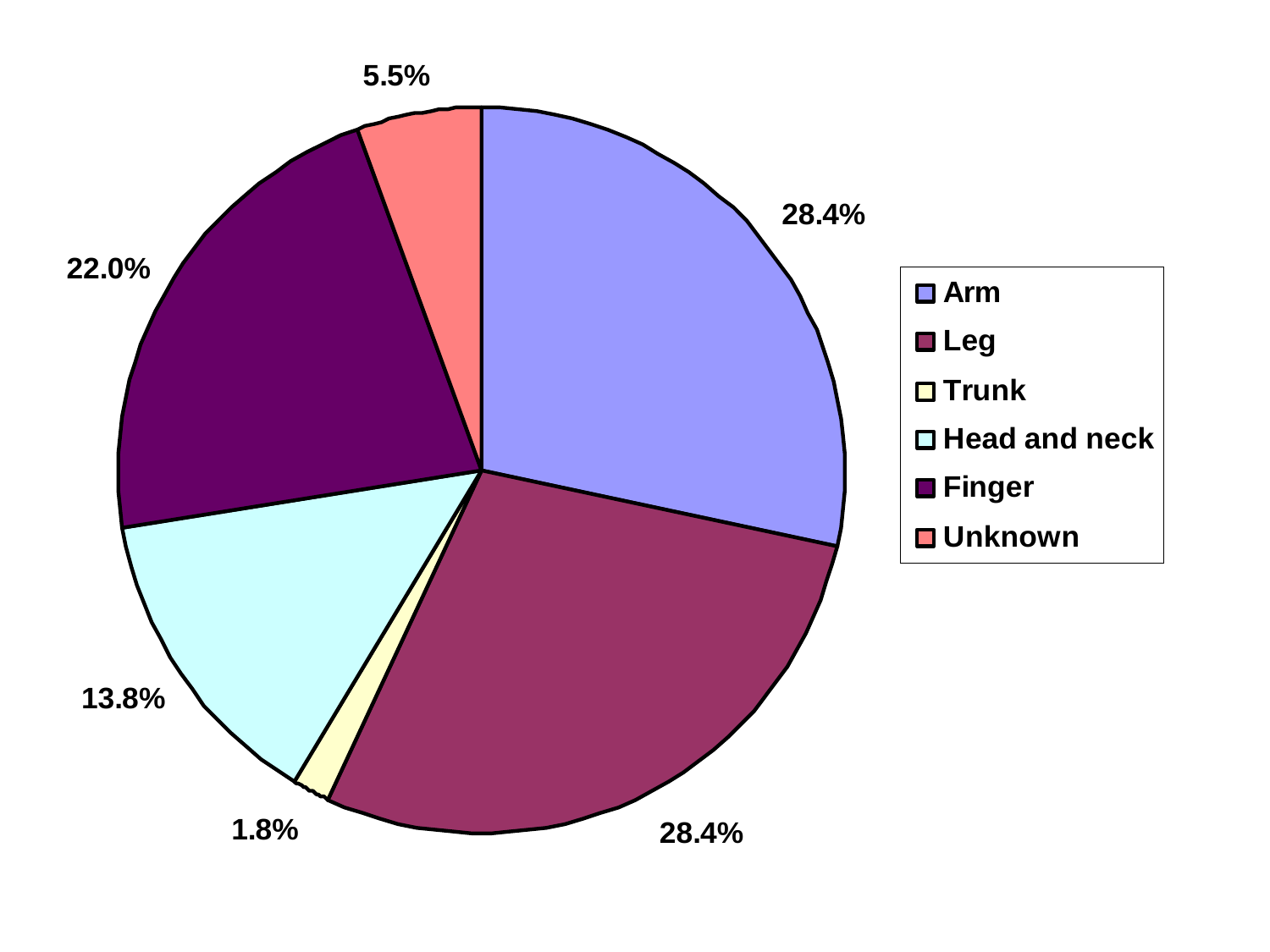

Supplement: Additional file 3 — Figure S1 – Spatial distributions of rabies classified by the sites of lesion. [file 1471-2334-8-113-S3.ppt]
